# The index system for the implementation effect evaluation of water-saving renovation of key medium-sized irrigation districts: A case study

**DOI:** 10.1371/journal.pone.0296953

**Published:** 2024-01-23

**Authors:** Mingliang Jiang, Chengcai Zhang

**Affiliations:** School of Water Conservancy and Transportation, Zhengzhou University, Zhengzhou, Henan, China; National Technical University of Athens: Ethniko Metsobio Polytechneio, GREECE

## Abstract

Irrigation district plays an important role in China’s agricultural production. In recent years, China conducted many water-saving renovation construction projects of irrigation districts. However, the current implementation effect of irrigation district water-saving renovation has not been well-characterized. Comprehensive and systematic evaluation of the implementation effect of water-saving renovation in irrigation districts can provide scientific basis for further construction and management of irrigation districts. This study screened preliminary index system in four dimensions: preliminary work, completion of construction tasks, completion of planned investment, management and reform. Based on the statistical results of the questionnaire surveys and subsequently analyzed, fourteen key evaluation indicators were finally identified. Percentile system and expert evaluation method were then used to determine the assigned score of every indicator. Based on the evaluation criteria, the evaluation method of the implementation effect was formulated. Ten key medium-sized irrigation districts in southeastern China were taken as a research example in this study, with the implementation effect of water-saving renovation of 10 irrigation districts being comprehensively evaluated. The results show that these irrigation districts have a relatively high implementation effect of water-saving renovation. The data results from the scores of four dimensions and fourteen indicators show that the index system is reasonable and practicable, and the evaluation results are basically in line with actual situation. These findings have a good practical reference value for making decisions about how to instruct the modernization development of water-saving renovation of irrigation districts in China.

## 1. Introduction

Irrigation district refers to an area or region with reliable water source, diversion, transmission and distribution channel system and corresponding drainage ditches. Irrigation district is the product of human economic activities, and it develops with the development of social economy. According to the designed irrigation area, irrigation districts could be divided into three types: large-sized, medium-sized and small-sized [1]. As a major public interest infrastructure, irrigation district is an important guarantee for water safety in China’s economy and society. The per unit area yield of grain of large and medium-sized irrigation districts is higher than the national average, which strongly supports the national food production security. The infrastructure in irrigation districts in China was mostly built in the 1950s to 1970s, most of them have been seriously damaged and lack a better resilience [2]. It is known that the resilience performance of critical water infrastructure plays an important role in emergency management and resilience policy-making [3, 4]. In 1998, China started the large-sized and medium-sized irrigation district water-saving renovation construction project, which is an effective measure for the management of water resources [5, 6]. The purpose is to solve the problem of aging of canal system and building engineering, improve the water use efficiency and the ability to resist disasters in irrigation district. However, irrigation district water-saving renovation involves many factors and large investment. Whether the implementation scheme is economical and reasonable, and whether the engineering construction is effective, requires scientific and comprehensive evaluation and analysis [7], which provides the basis for the decision-making department to formulate reasonable management measures and water-saving measures of irrigation districts.

At present, the evaluation methods for irrigation districts include comprehensive integration weighting method, variable fuzzy set theory method, extension evaluation method, artificial neural network method, analytic hierarchy process and fuzzy mathematics theory method, grey correlation method, TOPSIS comprehensive evaluation based on entropy weight, etc [8–12]. These methods have been applied to the comprehensive benefit evaluation of irrigation districts for many times. Although the above methods have certain guiding significance, the multi-linear form of the fuzzy comprehensive evaluation model can’t well reflect the indicators that need to be highlighted in the reality of the irrigation district, thus reducing the accuracy of the evaluation results. Grey correlation method and artificial neural network model need the level standard of irrigation district operation condition in calculation, which has not been formed in China at present, and the calculation results are difficult to apply in reality. The calculation of AHP model and linear interpolation method is relatively simple, but the evaluation indexes are subjective and the evaluation results are biased. The evaluation of water-saving renovation of irrigation districts in a region involves many factors such as agriculture, water conservancy, management, economic benefits and natural conditions. Moreover, the types of irrigation districts are complex, planting structures and irrigation methods are different, resulting in different economic benefits. In the process of establishing evaluation indexes and weights, some data will inevitably be lost, which will bring many difficulties and inapplicability to the comprehensive benefit evaluation of irrigation districts.

At home and abroad, there have been many research achievements in the evaluation of irrigation districts and irrigation infrastructure [13–18]. At first, people paid attention to the evaluation of the performance and economic benefits of irrigation districts, mainly including the operation status, water use efficiency, production efficiency, etc. With the increasingly prominent ecological and social problems and the proposition of sustainable development, researchers began to pay attention to the impact of the development of irrigation districts on the regional society, ecological environment and sustainable development. However, only a small number of researches were being implemented on the implementation effect evaluation of irrigation district water-saving renovation. The high quality water-saving renovation construction is the guarantee of bringing benefits into play in irrigation districts [19]. Therefore, it is essential and important to research and propose a scientific and rational method for assessing the implementation effect of water-saving renovation of key medium-sized irrigation districts. To close this gap, this paper aims to provide a method for evaluating the implementation effect of water-saving renovation of key medium-sized irrigation district in order to offer the base of water policy formulation and adjustment.

## 2. Research objective and methodology

### 2.1. Research objectives

This study aims to propose a rational method to assess the implementation effect of water-saving renovation of key medium-sized irrigation districts in order to provide support for policy-making and to guarantee that irrigation district can function well. The research contents are as follows: (1) determining the index system for the implementation effect evaluation of water-saving renovation of key medium-sized irrigation districts; (2) providing assessment method and criteria for the implementation effect; and (3) testing the index system and evaluation method based on a case study, through comparing evaluation results with actual situation to prove the reasonableness of the method.

### 2.2. Methodology

Questionnaire surveys, analytic hierarchy process (AHP), expert consultation method and case studies are popularly used approaches for searching or identifying assessment indicators [20, 21]. Qualitative methods and cases studies are more suitable for studying new issues than quantitative methods, for they offer more precise and detailed interpretations for new issues [22]. In the evaluation of irrigation districts construction and management, case study and survey are prove to be the elementary ways for data-gathering.

According to the former researches, a basic evaluation index system was set up with applying the theory and practice about irrigation districts construction and sustainable development from four dimensions: preliminary work, completion of construction tasks, completion of planned investment, management and reform. Twenty indicators were chose based on the relevant literature survey and expert interview. In addition, a questionnaire survey was conducted to ensure the reasonableness of the basic evaluation index system. These measures assured that the final evaluation index system would be comprehensive and credible. In the process of formulating an evaluation method, hundred mark system and expert consultation method were then used to determine the weight score of every indicator.

Weight score determination is one of the most important parts for assessing implementation effect of water-saving renovation of key medium-sized irrigation districts. AHP is a widely used policy-making method with multi-criteria. It was developed by Saaty in the 1970s to solve complicated decision matters, with catching both objective and subjective evaluation indicators [23]. This research drew lessons from the basic idea of AHP and applied it to the evaluation of irrigation districts.

**Fig 1:**
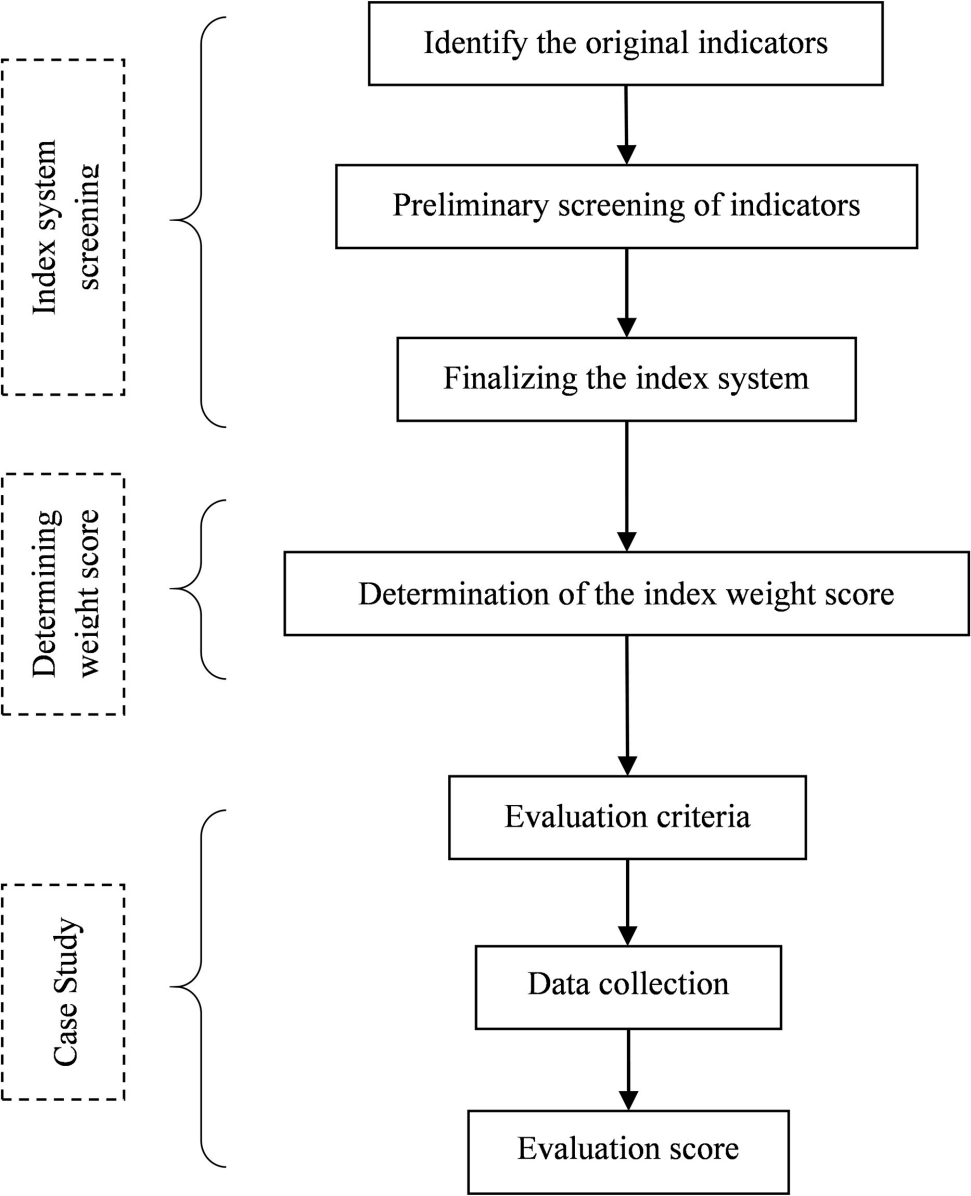
Methodology adopted in this study.

Based on the evaluation index system and evaluation method, followed by collecting the relevant research data, a case study was conducted to verify whether the evaluation results are in line with actual situation. The methodology adopted in this study is shown in Fig 1 [24].

## 3. Index system screening

### 3.1. Preliminary screening of indicators

The preliminary screening of indicators was based on whether they reflect the implementation effect of water-saving renovation of key medium-sized irrigation districts. It is essential to choose and include the indicators that can probably influence the implementation effect. So as to build a comprehensive evaluation index system, the reasonable evaluation of the implementation effect should be considered from diverse aspects. The water-saving renovation of irrigation district refers to the reconstruction, expansion and improvement of irrigation drainage facilities and auxiliary equipment, as well as the reform of the management system and operation mechanism of the irrigation district. Its purpose is to reduce water loss and improve the irrigation water use efficiency. During the whole process of renovation, construction task is a critical part. Therefore, the completion level of construction task is an important criterion to assess the implementation effect of water-saving renovation. Besides, the water-saving renovation is a construction project, it involves planning scheme, capital investment, and management and reform. Tian et al. proposed that the comprehensive evaluation of implementation effect of water-saving renovation in large-sized irrigation districts mainly include construction process standardization, completion of construction task and investment, economic benefits, management and reform, and sustainable development [25]. This paper identified the index system using the four dimensions: preliminary work, completion of construction tasks, completion of planned investment, management and reform.

The following three approaches are conducted for indicators choice. First, relevant literature analysis and official policy study were used to pick out the original indicators. Secondly, preliminary indicators were chose on the basis of the expert interview method. The final index system was determined through the questionnaire survey method. Original indicators related to the implementation effect of water-saving renovation of key medium-sized irrigation districts were identified. Based on literature analysis and policy study, twenty-one relevant papers and fifteen recent government policies were contained. Then, thirty-one relevant original indicators were found. But, there were some similarities among the thirty-one original indicators, so interviews were implemented to determine the applicability of original indicators. Seven relevant scholars and ten government regulators of irrigation district construction were invited to participate in the interview. Eventually, the four dimensions were approved, and the original indicators were reduced to eighteen preliminary indicators (Table 1), which were considered to be more suitable represent the implementation effect of water-saving renovation of key medium-sized irrigation districts.

**Table 1:** Preliminary index system of implementation effect evaluation.

Target Layer	Dimension Layer	Index Layer
**Implementation effect of water-saving renovation of key medium-sized irrigation districts**	Preliminary work (A1)	Completion of implementation plan (A11); Project commencement (A12).
	Completion of construction tasks (A2)	Construction of head works (A21); Construction of canals and ditches (A22); Construction of canal and ditch structures (A23); Completion of engineering management and protection facilities (A24); Completion of metering facilities (A25); Acceptance of engineering works (A26); Engineering quality (A27).
	Completion of planned investment (A3)	Completion of central financial investment (A31); Completion of central financial investment by the end of December 2019 (A32); Local counterpart funds arrival rate (A33).
	Management and reform (A4)	Whether to implement the comprehensive reform of agricultural water price (A41); Proportion of executed water price in operation cost (A42); Implementation rate of personnel funds (A43); Implementation rate of maintenance funds (A44); Water fee collection rate (A45); Education level of management personnel (A46).

### 3.2. Finalizing the index system

#### 3.2.1. Questionnaire survey

The questionnaire was designed to examine the preliminary indicators, particularly from the aspect of rationality and practicality. The questionnaires include eighteen indicators with a 5-point Likert scale (1—can be ignored or not important; 2—somewhat important; 3—important; 4—very important; and 5—extremely important) [26]. Five-hundred survey questionnaires were distributed to personnel in irrigation district management departments as well as to government regulators of water resource and scholars of research institution. They were asked to make their own judgments on the eighteen indicators according to their irrigation district construction and management experience. This survey was conducted from July to September 2020. Finally, 362 completed responses were received. The respondent rate was 72.4%, in line with the standard for questionnaire surveys in investigation and research.

#### 3.2.2. Mean value and ranking of the indicators

The scores and rankings of the eighteen indicators of implementation effect of key medium-sized irrigation districts water-saving renovation were examined by descriptive statistics (Table 2). The mean values for these eighteen indicators range from a minimum of 2.238 (A45) to a maximum of 4.615 (A22). Half of the indicators’ mean values are over 4.00 (9 indicators), with just four being below 3.0 (A27, A33, A45, and A46). This indicates that most of the indicators are quite important and can be used as a representation of the implementation effect of irrigation districts water-saving renovation in China. Of the top five indicators with the highest mean values, three belong to the completion of construction tasks dimension (A2). The result shows that the completion of construction tasks has stronger influences in irrigation districts water-saving renovation.

**Table 2:** Scores and ranking of the indicators.

Dimension	Indicator	Mean	Rank	Group Mean	Group Rank	Verification
**Preliminary work (A1)**	A11	4.413	4	4.135	2	Pass
	A12	3.856	11			Pass
**Completion of construction tasks (A2)**	A21	4.589	2	4.358	1	Pass
	A22	4.615	1			Pass
	A23	4.229	7			Pass
	A24	4.558	3			Pass
	A25	4.148	8			Pass
	A26	4.008	9			Pass
	A27	2.814	15			No
**Completion of planned investment (A3)**	A31	3.246	14	3.560	4	Pass
	A32	3.873	10			Pass
	A33	2.673	16			No
**Management and reform (A4)**	A41	4.255	6	3.945	3	Pass
	A42	3.437	13			Pass
	A43	4.332	5			Pass
	A44	3.756	12			Pass
	A45	2.238	18			No
	A46	2.364	17			No

#### 3.2.3. The final index system

According to the analysis of questionnaire results, the most of indicators have been proven to be effective. In this study, the indicators with mean values below 3.0 were eliminated, namely engineering quality (A27), local counterpart funds arrival rate (A33), water fee collection rate (A45) and education level of management personnel (A46), which indicated that these four indicators are relatively less important for assessing the implementation effect of water-saving renovation of key medium-sized irrigation districts with regards to reasonability and operability. The final composition of the index system is shown in Table 3.

**Table 3:** The final index system of implementation effect evaluation.

Target Layer	Dimension Layer	Index Layer
**Implementation effect of water-saving renovation of key medium-sized irrigation districts**	Preliminary work (A1)	Completion of implementation plan (A11); Project commencement (A12).
	Completion of construction tasks (A2)	Construction of head works (A21); Construction of canals and ditches (A22); Construction of canal and ditch structures (A23); Completion of engineering management and protection facilities (A24); Completion of metering facilities (A25); Acceptance of engineering works (A26).
	Completion of planned investment (A3)	Completion of central financial investment (A31); Completion of central financial investment by the end of December 2019 (A32).
	Management and reform (A4)	Whether to implement the comprehensive reform of agricultural water price (A41); Proportion of executed water price in operation cost (A42); Implementation rate of personnel funds (A43); Implementation rate of maintenance funds (A44).

## 4. Case study

### 4.1. Study area

In this study, 10 key medium-sized irrigation districts in southeastern China were taken as research examples, these irrigation districts located in a province. The province is located in the lower reaches of the Yangtze and Huai River basins There are 264 medium-sized irrigation districts in the province, which undertake about 42% of the whole province’s farmland irrigation work.

In 2019, 10 key medium-sized irrigation districts in this province implemented water-saving renovation construction projects, involving 10 counties, with total designed irrigation area of 1176.73 km^2^ and total effective irrigation area of 1000.60 km^2^. After the implementation of these projects, not only did it solve the safety hazards and improve the safe operation ability, but also the benefits of water conservation and agricultural yield increase were significant, and the guarantee ability of irrigation districts for agricultural production was significantly improved.

### 4.2. Evaluation criteria

So as to conduct a scientific and reasonable evaluation of the implementation effect of water-saving renovation, it is urgent to build the evaluation criteria [27]. Standard evaluation criteria are determined on a scientific basis, which should reasonable and easy to operate [28]. In this study, the evaluation criteria of the water-saving renovation of key medium-sized irrigation districts implementation effect were set according to percentile system. Assigned score, namely full score of each indicator in the index system were determined by experts and government water resources department in a quantitative manner, and the sum of all indicator’s assigned score is equal to 100. All indicators are quantitative indicators and have a definite score calculation method. The score represent the implementation effect of each indicator. The evaluation criteria of the water-saving renovation of key medium-sized irrigation districts implementation effect for each indicator are shown in Table 4.

**Table 4:** Evaluation criteria of index system.

Index	Assigned score	Score calculation method
**A11**	5	If the implementation scheme is approved within the time required by the provincial water conservancy department, 5 points will be obtained; otherwise, 0 point will be obtained; If the provincial water conservancy department has no requirements, 5 points will be given if it is completed before the end of June 2019, and 0 point will be given if it is later than June 2019.
**A12**	5	5 points will be given if the construction starts before the end of October 2019, and 0 point will be given if it is later than October 2019.
**A21**	6	According to the percentage of the total number of actually completed headworks to the planned number.
**A22**	20	Percentage of the actual completed canal (ditch) reconstruction length to the planned value.
**A23**	10	Percentage of actually completed canal (ditch) system buildings (sets) in the planned number.
**A24**	2	According to the percentage of the number of actually completed management and protection facilities in the planned number.
**A25**	2	The percentage of the total number of measuring facilities (set) actually completed to the planned number.
**A26**	10	10 points will be given if the completion acceptance is completed, 5 points will be given if the completion acceptance is only completed, and 0 point will be given if none is completed.
**A31**	10	(Completed central investment amount/planned central investment amount of implementation plan) × 100%, scored proportionally.
**A32**	10	(The central investment amount/the central investment amount of the implementation plan will be completed by the end of December 2019) × 100%, ≥ 80%, 10 points will be given, and scores will be given proportionally when < 80%.
**A41**	4	4 points for "Yes" and 0 point for "No".
**A42**	4	If the proportion is ≥ 100%, 4 points will be obtained, and the period will be scored proportionally when < 100%.
**A43**	6	If the proportion is ≥ 100%, 6 points will be obtained, and the period will be scored proportionally when < 100%.
**A44**	6	If the proportion is ≥ 100%, 6 points will be obtained, and the period will be scored proportionally when < 100%.

### 4.3. Data collection

In 2019, there are 10 irrigation districts in the province conducted water-saving renovation construction projects, namely Irrigation District 1 (IrrD1), Irrigation District 2 (IrrD2), Irrigation District 3 (IrrD3), Irrigation District 4 (IrrD4), Irrigation District 5 (IrrD5), Irrigation District 6 (IrrD6), Irrigation District 7 (IrrD7), Irrigation District 8 (IrrD8), Irrigation District 9 (IrrD9) and Irrigation District 10 (IrrD10).

According to the evaluation index system and evaluation criteria above, the required relevant data of the 10 key medium-sized irrigation districts were collected. All the data have been checked without error.

### 4.4. Calculation results of evaluation score

According to the evaluation criteria and the data collected, the score for each indicator of the 10 key medium-sized irrigation districts was calculated. For the indicator A21 to A25 of completion of construction tasks, some irrigation districts may don’t have the plan task of construction, so the dimension score *SC*(2) is calculated according to Eq (1) based on weighting method. Finally, the target evaluation score *SC*_*k*_ of irrigation district IrrD*k* is calculated according to Eq (2). The results are shown in Table 5.

$$SC\left(2\right)=\sum_{i=2,j=1}^{i=2,j=5}SC\left(ij\right)\times 40÷\sum_{i=2,j=1}^{i=2,j=5}SC{\left(ij\right)}_{A}$$ (1)

$$SC_{k}=SC\left(2\right)+SC\left(26\right)+\sum_{i\neq 2}SC(ij)$$ (2)

where *SC*(*ij*) represents the score of index A*ij*, *SC*(*ij*)_*A*_ represents the assigned score of index A*ij* which have the plan task of construction.

**Table 5:** Calculated results of irrigation districts implementation effect.

Irrigation District	Evaluation Score of Indicator														*SC*_*k*_
	A11	A12	A21	A22	A23	A24	A25	A26	A31	A32	A41	A42	A43	A44	
**IrrD1**	0.00	5.00	-	20.00	10.00	-	2.00	5.00	10.00	10.00	4.00	4.00	6.00	6.00	90.00
**IrrD2**	0.00	5.00	6.00	18.66	10.00	-	2.00	5.00	10.00	10.00	4.00	4.00	6.00	6.00	88.59
**IrrD3**	0.00	5.00	-	20.00	10.00	-	2.00	10.00	10.00	10.00	4.00	4.00	6.00	6.00	95.00
**IrrD4**	0.00	5.00	6.00	20.00	10.00	-	2.00	10.00	10.00	10.00	4.00	4.00	6.00	6.00	95.00
**IrrD5**	5.00	5.00	6.00	-	10.00	-	2.00	5.00	10.00	10.00	4.00	4.00	6.00	6.00	95.00
**IrrD6**	5.00	0.00	6.00	20.00	10.00	-	2.00	10.00	10.00	10.00	4.00	4.00	6.00	6.00	95.00
**IrrD7**	0.00	5.00	-	20.00	10.00	2.00	2.00	10.00	10.00	10.00	4.00	3.20	6.00	6.00	94.20
**IrrD8**	0.00	5.00	-	20.00	10.00	-	2.00	10.00	10.00	10.00	4.00	1.44	6.00	6.00	92.44
**IrrD9**	0.00	0.00	-	19.75	10.00	-	-	10.00	10.00	10.00	4.00	4.00	6.00	6.00	89.67
**IrrD10**	0.00	5.00	6.00	20.00	10.00	-	2.00	5.00	10.00	10.00	4.00	4.00	6.00	6.00	90.00

Note: “-” refers to don’t have the plan task of construction.

## 5. Results and discussion

**Fig 2:**
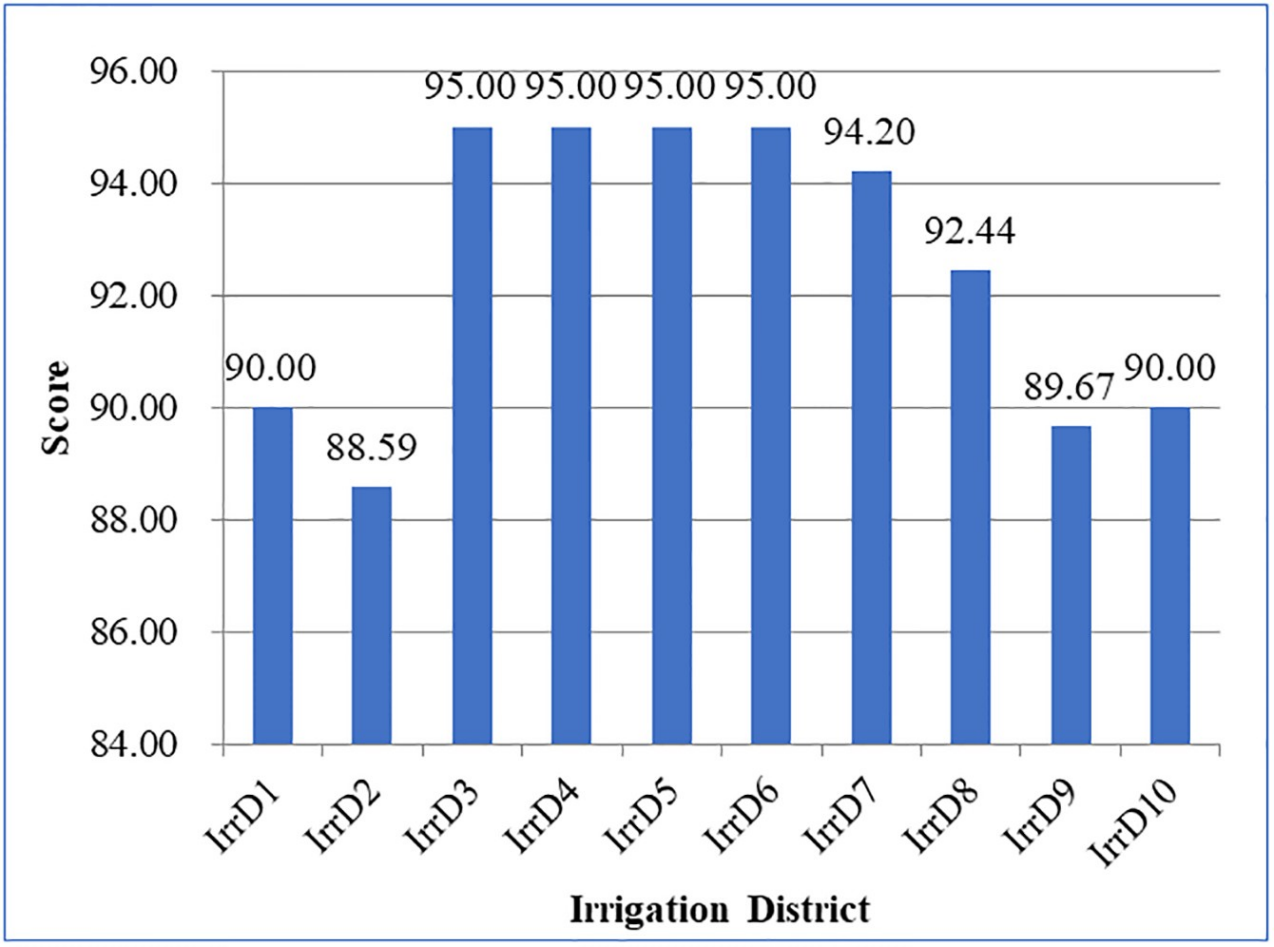
Evaluation scores for the implementation effect of 10 irrigation districts.

As a case study, 10 irrigation districts in southeastern China were used as research examples for examining the validity of the proposed index system and evaluation method for the implementation effect of water-saving renovation of key medium-sized irrigation districts in this study. The scores for the implementation effect of the 10 irrigation districts renovation construction were obtained. Evaluation scores of the 10 irrigation districts are illustrated in Fig 2. It was discovered that, evaluation score of all 10 irrigation districts are higher than 85.00, showed that the implementation effect was relatively excellent. Four irrigation districts achieved the same top score (IrrD3, IrrD4, IrrD5, IrrD6, 95.00), while one irrigation district received the lowest score (IrrD2, 88.59). The mean evaluation score of 10 irrigation districts is 92.49, and five irrigation districts had the evaluation score above mean value.

**Fig 3:**
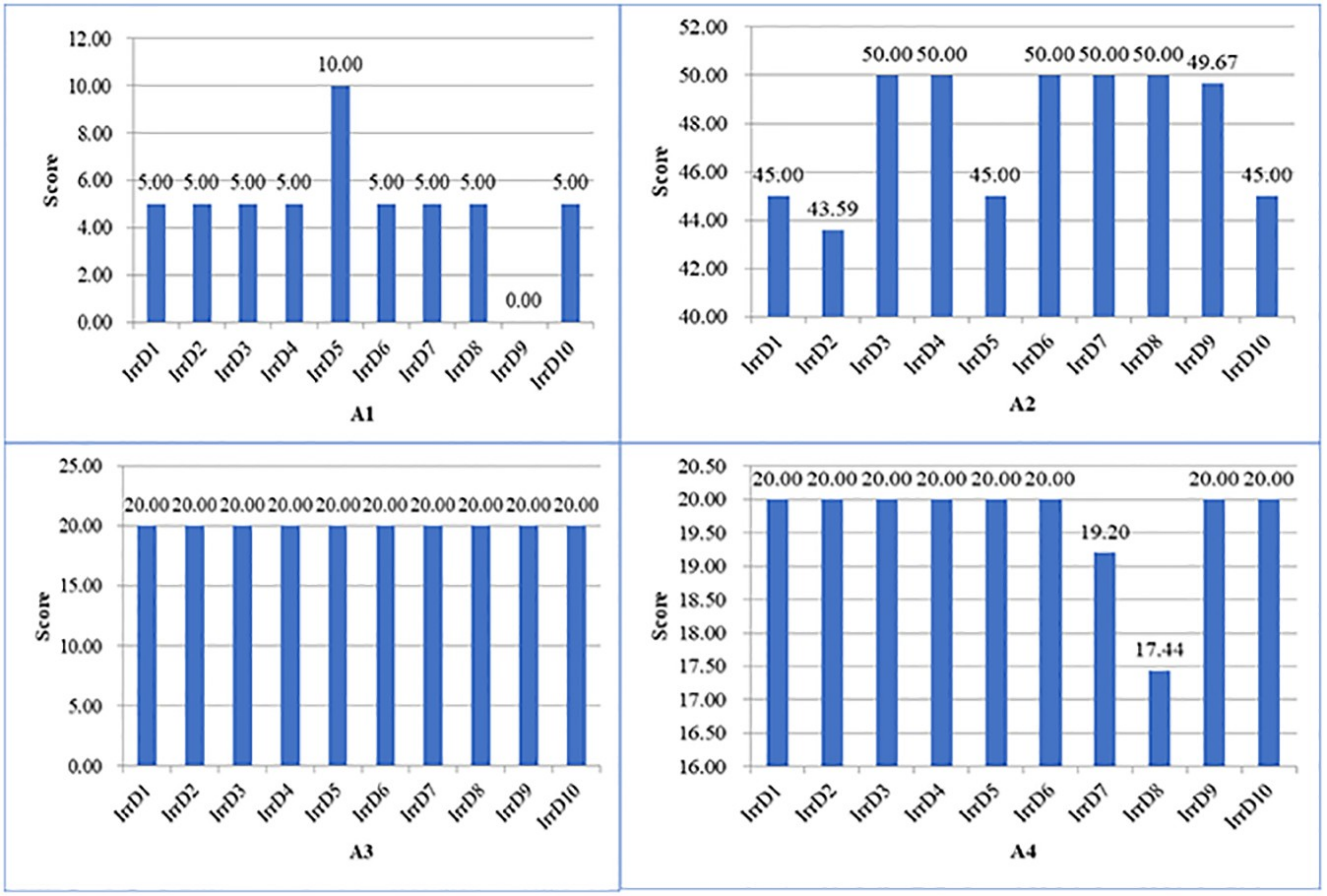
Scores for the four dimensions for the implementation effect of 10 irrigation districts.

**Fig 4:**
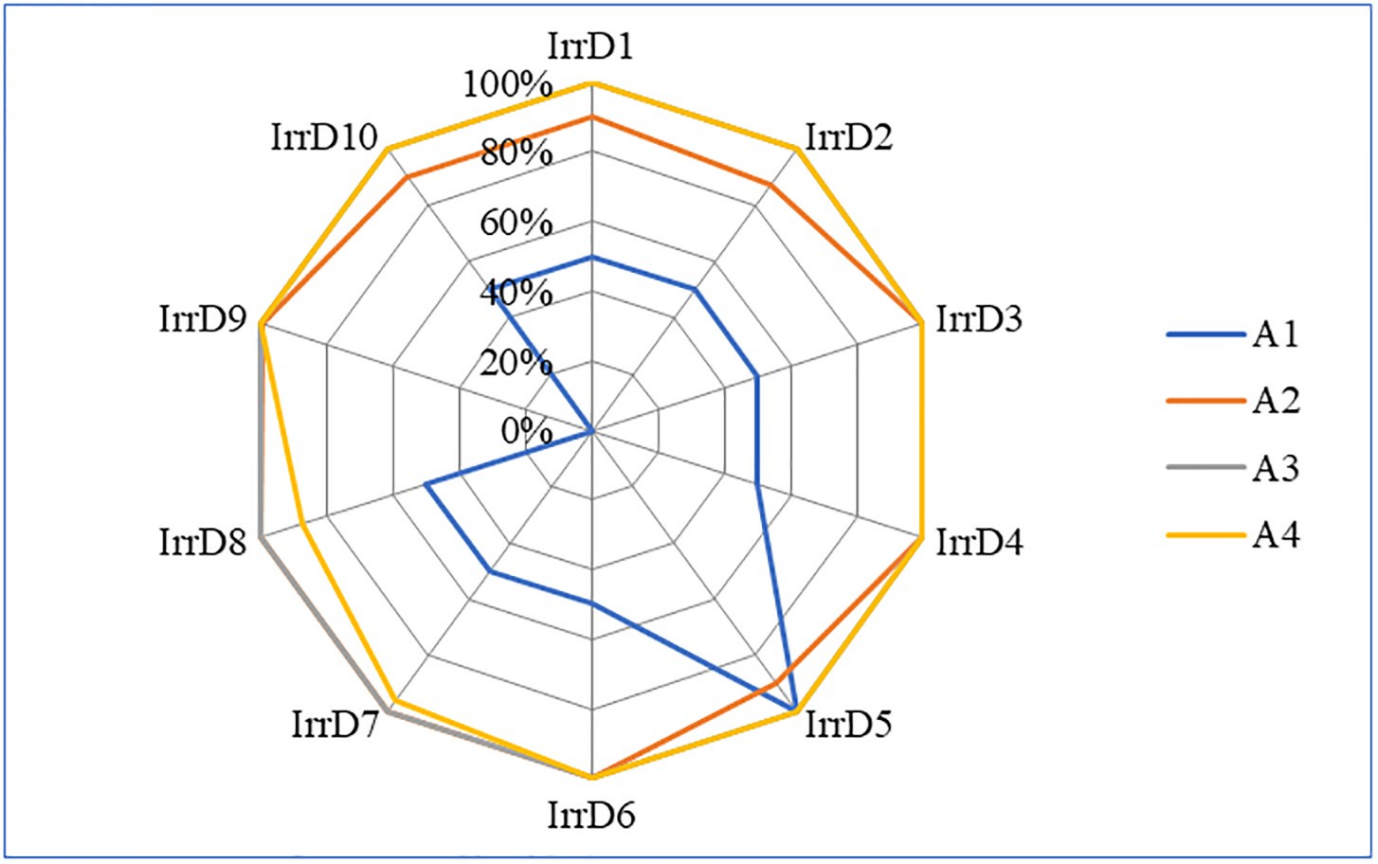
Score rate for the four dimensions of 10 irrigation districts.

Evaluation scores and score rate for the four dimensions of the 10 irrigation districts are illustrated in Figs 3 and 4, respectively. It was discovered that, in the preliminary work dimension, only one irrigation district gained full score (IrrD5, 10.00), most of the irrigation districts had score of 5.00, it showed that these irrigation districts didn’t doing well in time node of implementation plan approval and construction start. In the completion of construction tasks dimension, five irrigation districts received full score (IrrD3, IrrD4, IrrD6, IrrD7, IrrD8, 50.00), and all 10 irrigation districts had the evaluation score above 40.00, it should that most of planned engineering construction tasks have been completed practically. In addition, some irrigation districts don’t have plan of construction tasks about some indicators. In the completion of planned investment dimension, all 10 irrigation districts reached full score 20.00, it showed that the use efficiency of central government investment funds was relatively high. One possible reason is that government usually supervises and requires the acceleration of the execution progress of central investment funds in China. In the management and reform dimension, only two irrigation districts didn’t gained full score (IrrD7, 19.20; IrrD8, 17.44), it was caused by evaluation score of indicator A42, so these two irrigation districts should raise the executed water price properly to reflect the precious value of agricultural water resources.

According to the data materials analysis of these 10 irrigation districts in 2021 and expert on-site research survey, it is believed that the construction process of the water-saving renovation construction projects is standardized, the construction tasks and investment completion are relatively good, and the management and reform are effective. This proves that the evaluation results achieved using the evaluation method is basically consistent with the actual situation of the water-saving renovation in the irrigation districts.

When comparing with other similar studies, the evaluation index system and method proposed in this study is reasonable and practicable, and relevant data of irrigation districts is easy to obtain. Because of the large number of evaluation indicators and some indicator data is hard to access or be accurate, the index system built by Tian et al. is complicated [25], And, its evaluation method and process are more cumbersome. Also, in some studies, there are many methods for comprehensive evaluation, such as fuzzy comprehensive evaluation, artificial neural network evaluation, and extension evaluation. However, these methods are often limited to reflecting a certain type of uncertainty in the evaluation indicators, and cannot balance randomness and fuzziness while reflecting actual indicators [29].

## 6. Conclusions

Most prior studies on irrigation district water-saving renovation concentrated on economic benefits analysis, agricultural production output and ecological environment, with a limited number of studies aiming to assess the implementation effect of water-saving renovation. To address this knowledge gap, a method for evaluating the implementation effect of water-saving renovation of key medium-sized irrigation districts was proposed.

This study builds an evaluation index system including four dimensions and fourteen critical indicators for the implementation effect of key medium-sized water-saving renovation projects in irrigation districts. The selected evaluation indicators cover aspects such as the preliminary work, completion status of construction tasks, completion of planned investment, management and reform of irrigation districts. They can comprehensively reflect the implementation effect of key medium-sized irrigation districts water-saving renovation, and the data of evaluation indicators is easy to obtain and calculate. Then validated the evaluation index system for 10 key medium-sized irrigation districts in southeastern China. The results show that these 10 irrigation districts have a relatively high implementation effect in general, however, some scores of dimensions and indicators are not ideal. To promote the development of irrigation district modernization, emphases may be placed on taking effective measures and encouraging policies in implementation scheme preparation and approval, commencement of construction works. Meanwhile, it is necessary to complete acceptance of engineering works as soon as possible under compliance with regulations circumstances. The research data from scores of four dimensions and fourteen indicators show that the evaluation index system is practicable, with evaluation results in line with actual situation. Although the evaluation index system proposed here can offer a beneficial framework for researchers to further assess the implementation effect of irrigation districts water-saving renovation, the study is just a primary exploration that is open to examine and improvement. A follow-up research based on more related indicators is expected to test the usability of evaluation method and index system proposed in this paper.

In further research, it is essential to expand and widen the evaluation index system with the fast development of irrigation district modernization, such as sustainable development dimension, local financial support, and negative issues of engineering quality and safety accidents. In addition, the relationship between the different indicators of index system should be considered.

## Supporting information

S1 Data (XLSX)
